# Correction to “Intratumoral *Fusobacterium nucleatum* Drives Cancer‐Associated Fibroblasts Enrichment and Immune Exclusion in Esophageal Squamous Cell Carcinoma”

**DOI:** 10.1002/ags3.70253

**Published:** 2026-07-21

T. Ofuchi , K. Shinjo , Q. Hu , et al., “Intratumoral *Fusobacterium nucleatum* Drives Cancer‐Associated Fibroblasts Enrichment and Immune Exclusion in Esophageal Squamous Cell Carcinoma,” Annals of Gastroenterological Surgery 10, no. 2 (2025): 611–620. 10.1002/ags3.70116 41799583 PMC12962025

The author list for the above article was incorrect. Dr. Yutaka Kondo and Dr. Keiko Shinjo should be added as co‐authors. The final author list is as follows:

Takashi Ofuchi^1,2^, Keiko Shinjo^3^, Qingjiang Hu^1^, Kazuki Omachi^1,2^, Kosuke Kanemitsu^2^, Hajime Otsu^1^, Yusuke Yonemura^1^, Taro Tobo^4^, Yoshifumi Baba^5^, Masaaki Iwatsuki^2^, Yutaka Kondo^3^, and Koshi Mimori^1^.

^1^Department of Surgery Beppu Hospital, Kyushu University, Oita Japan

^2^Department of Gastroenterological Surgery Graduate School of Medical Sciences, Kumamoto University, Kumamoto Japan

^3^Division of Cancer Biology, Nagoya University Graduate School of Medicine, 65 Tsurumai‐cho, Showa‐ku, Nagoya, Japan

^4^Department of Pathology Beppu Hospital, Kyushu University Oita Japan

^5^Department of Gastrointestinal Surgery Graduate School of Medicine, The University of Tokyo, Tokyo Japan

The online article has been corrected.

We apologize for this error.

